# Prevalence of non-communicable diseases among HIV positive patients on antiretroviral therapy at joint clinical research centre, Lubowa, Uganda

**DOI:** 10.1371/journal.pone.0221022

**Published:** 2019-08-09

**Authors:** Sheila Kansiime, Doris Mwesigire, Henry Mugerwa

**Affiliations:** 1 College of Health Sciences, Makerere University, Kampala, Uganda; 2 MRC/UVRI Uganda Research Unit on AIDS, Entebbe, Uganda; 3 Joint Clinical Research Centre, Luboowa, Uganda; NPMS-HHC CIC / LSH&TM, UNITED KINGDOM

## Abstract

**Introduction:**

Antiretroviral therapy (ART) has changed the course of HIV/AIDs by enabling patients to live longer, raising concern of the co- existence of HIV with other chronic illnesses, notably non-communicable diseases (NCDs). NCDs are on the rise in developing countries and evidence shows higher occurrence among people living with HIV (PLHIV). In Uganda, the burden of NCDs among PLHIV remains largely unquantified.

**Objective:**

To determine the prevalence of hypertension, osteoporosis, diabetes mellitus, renal impairment, asthma, cardiomyopathy and multi-morbidity among HIV positive patients, receiving Anti-Retroviral Therapy at Joint Clinical Research Centre, Lubowa, Uganda.

**Methods:**

This was a cross-sectional study conducted among 387 systematically sampled patients, receiving ART at the Joint Clinical Research Centre, Lubowa, between March and April 2017. The study used data extracted from routine care patient files to identify individuals with non-communicable diseases. Prevalence of the NCDs was estimated and reported with 95% confidence intervals. Prevalence was also reported at various levels of socio- demographic, behavioural and clinical factors.

**Results:**

The overall prevalence of having at least one NCD was 20.7% (95% CI: 16.7–24.5). The prevalence of hypertension was 12.4% (95% CI: 9.1–15.7), osteoporosis 6.5% (95% CI: 4.0–8.9), diabetes mellitus 4.7% (95% CI: 2.6–6.8), renal impairment 1.6% (95% CI: 0.3–2.8), asthma 1.6% (95% CI: 0.3–2.8), and cardiomyopathy 1.3% (95% CI: 0.2–2.4). Prevalence of multi-morbidity was 4.7% (95% CI: 2.6–6.8). Prevalence was significantly higher among older participants, widowed participants and individuals with an opportunistic infection.

**Conclusion:**

Non-communicable diseases are common among people living with HIV. There is need to encourage early diagnosis and treatment of non-communicable diseases in PLHIV in Uganda.

## Introduction

Human Immunodeficiency Virus (HIV) and Acquired Immune Deficiency Syndrome (AIDS) remain a major public health concern that disproportionately affects sub-Saharan Africa (SSA).

Of the 36.9 million people living with HIV worldwide, it is estimated that nearly 70% live in sub Saharan Africa [1]. Eastern Africa is the second most affected region in Africa after southern Africa [2] which together are estimated to have over 17.7 million people living with HIV [1]. In Uganda, the prevalence of HIV is estimated to be 7.3%, the highest in the East African region [3].

In the early 80’s and 90’s, HIV/ AIDS was a mysterious, feared, untreatable and often fatal condition [4]. However, the advent of antiretroviral therapy (ART) has changed the course of the disease, giving patients and scientists new hope for fighting it and living longer [5].

Conversely, the aging cohort of people living with HIV (PLHIV) raises concern of the coexistence of HIV with other chronic illnesses, notably the non-communicable diseases (NCDs) [6]. According to WHO 2015, it is estimated that NCDs are the cause of death of 38 million people each year, and three quarters of these occur in low and middle income countries (LMICs) which are already burdened by HIV [7].

More so, people living with HIV have been found to have a higher risk of having non communicable diseases. This is a result of the HIV infection, antiretroviral therapy, HIV related immunosuppression, increasing age of PLHIV, HIV related inflammation, in addition to traditional risk factors of NCDs such as: tobacco smoking, alcohol use, physical inactivity, unhealthy diets, and the demographic and epidemiologic transitions [8].

Although evidence from high-income countries is definitive as to the emerging importance of NCDs for PLHIV, there are far fewer data and research advances regarding such conditions in LMICs [6]. In Uganda, the burden of NCDs among PLHIV remains largely unquantified.

This study aimed to describe the prevalence of hypertension, osteoporosis, diabetes mellitus, renal impairment, asthma, cardiomyopathy and multi-morbidity, among HIV positive patients receiving ART at JCRC, Lubowa Uganda.

## Materials and methods

### Study design

This was cross-sectional study conducted between March and April 2017. It was part of a larger study that was investigating the association between NCDs and quality of life (measured by the WHO Quality of life Bref), in addition to identifying challenges faced in accessing health care for NCDs.

### Study site

The study was carried out at the outpatient department, Joint Clinical Research Centre (JCRC), located at Plot 101, Lubowa Estates, off Entebbe road, Kampala, Uganda. JCRC is a specialized HIV care centre that was established in 1990 as a clinical research facility to address challenges of HIV care and management in Uganda and create expertise in clinical research and treatment.

JCRC has been providing free anti-retroviral therapy since 1996, in addition to HIV counselling and testing services. According to the JCRC Quarterly report 2016 a total of 15,580 patients had visited Lubowa clinic for at least one clinical service in the preceding three months. Patients report every 3 months for antiretroviral therapy treatment refill. They come from areas around Lubowa, including Kampala, Wakiso, Entebbe. The patients who attend the facility are of different social economic statuses.

Information on NCDs is collected in routine care at the facility during patient visits every three months. Hypertension is screened for at every clinic visit, diabetes mellitus II, asthma, cardiomyopathy and osteoporosis are usually screened for when a patient complains of certain symptoms during the routine follow up visits. Renal impairment is screened for among patients upon being started on tenofovir. However, some NCDs are screened for as a result of participation in research projects that require screening for certain ailments / medical conditions.

### Population

#### Target population

People living with HIV/AIDs receiving ART at an urban specialized HIV care centre in Uganda.

#### Accessible population

PLHIV that attended JCRC for clinical review and/or drug re-supply during the study period.

### Study population

Inclusion criteria

- Individuals aged 16 and above, who had received ART for more than 2 months, and gave informed consent.

Exclusion criteria

- Individuals who were physically or mentally unable to participate.
- Individuals who were unable to comprehend either English or Luganda.

### Sample size

The Kish Leslie formula for descriptive studies (Kish, 1965) was used to estimate the required sample size to determine the prevalence of NCDs among people with HIV/AIDs receiving ART at the Joint Clinical Research Centre Lubowa, Uganda. A prevalence of 50% was used to estimate the maximum sample size required.

$$n=\frac{\left(Z^{2}\times P\times Q\right)}{d^{2}}$$

n: The required sample size

Z: standard normal value at 95% level of confidence (1.96)

P: Prevalence of the NCDs selected is unknown (assuming 50%)

d: Allowing an error of 5%

$$n=\frac{\left(1.96^{2}\times 0.5\times (1-0.5)\right)}{0.05^{2}}=385\;people$$

### Study variables

#### Outcomes of interest

- Hypertension, osteoporosis, diabetes mellitus, renal impairment, asthma, cardiomyopathy and multi-morbidity were the outcomes of interest. They were all treated as binary variables. An individual was categorized as having a condition or not.

#### Other variables

- Demographic factors: age, sex, marital status, level of education, occupation
- Other health related factors: WHO HIV stage, opportunistic infections
- Life style factors: smoking, alcohol use.

### Data collection

Research experienced personnel with bachelors in nursing and midwifery, already working within JCRC were recruited and trained as research assistants. They were responsible for the data extraction and interviews. Patients presenting to the study facility for clinical review and/or drug re-supply, were consented into the study.

Relevant information was then extracted from their patient files and entered into a data extraction form by the nurses. The patient files included information on a particular individual from when they were enrolled as patients at JCRC.

At the facility the following criterion is used to determine whether an individual has one of the above diseases:

Hypertension: If an individual is found to have a systolic blood pressure (SBP) of 140mm Hg or more, or a diastolic blood pressure (DBP) of 90 mm Hg or more, or taking antihypertensive medication.

Diabetes mellitus: Having a fasting blood sugar equal to or more than 140mg/dl (7.8mmol/l) on 2 or more occasions, or a Random blood sugar of more than 200mg/dl (11.8mmol/l) or having a history of diagnosis of diabetes mellitus.

Renal impairment: Having urea levels, creatinine levels or potassium levels above the normal range or a calculated Glomerular filtration rate (GRF) of less than 50ml/ min.

Asthma: This is assessed using medical history and proof of asthma relief medicines for example salbutamol inhaler and medical documents showing asthma treatment, clinical examination to confirm presence of rhonchi, spirometry to assess reversible airway obstruction with an FEV1 /FVC ratio less than 0.7 in an attack but with marked improvement on administration of bronchodilators that it: an increase in FEV1 (or FVC) of ≥12% and an absolute increase in FEV1 (or FVC) of ≥200mL.

Cardiomyopathy: A chest X-ray indicative of an enlarged heart; or a cardiac echo with a cardiologist report confirming a cardiomyopathy.

Osteoporosis: This is based on a clinical history of low velocity, atraumatic fractures or fragility fractures (hip, vertebrae, spine, forearm and wrist) and associated bone pain, taking medications for bone health, clinical examination and X-ray reports

### Data management

All study data was checked for accuracy, completeness and consistency at the end of each day by the PI, and any identified errors were corrected in real time.

Hard copies of the questionnaires were stored in a water proof box file and electronic data bases were kept in password protected computers. An external hard drive was used to back up the data.

Double data entry was done using Epi-data version 3.1.

### Data analysis

All data analysis was done using STATA version 13.0 (STATA, College Station, Texas, USA). All continuous variables were summarized using means and standard deviations or medians and inter-quartile ranges, while categorical variables were summarized using proportions or percentages. Prevalences were reported with 95% confidence intervals.

### Ethical consideration

Permission to proceed with the study was obtained from the Clinical Epidemiology Unit and the Joint Clinical Research Centre. Approval was obtained from; the Makerere University School of Medicine Research and Ethics Committee, and Uganda National Council for Science and Technology.

Written informed consent was obtained from study participants before they were enrolled into the study. Participant details such as names or phone numbers were not included in the data extracted from the patient files. Personal identification numbers were assigned to participants at enrolment and used in the questionnaires.

## Results

### Socio-demographic characteristics of study participants

387 participants were systematically selected from patients who attended the Joint Clinical Research Centre March to April 2017 to participate in the study. The median age of the participants was 42 years (range: 20–75years). Overall 66.1% of the participants were female, 47.8% were married or living together, 74.7% had attained Senior 4 or below level of education, and 58.4% reported being self-employed (Table 1).

**Table 1:** Socio-demographic characteristics of 387 participants at the Joint Clinical Research Centre, Lubowa, March to April 2017.

Variable	Number (N = 387)	Percentage (%)
**Age (categorized)**		
18–29	43	11.3
30–49	243	63.6
50–69	92	24.1
70 +	4	1
**Sex**		** **
Female	256	66.1
Male	131	33.9
**Marital status**		
Single	91	23.5
Married/Living together	185	47.8
Widowed	51	13.2
Divorced/Separated	60	15.5
**Education**		
No formal education	21	5.4
Primary	131	33.9
S1-S4	137	35.4
S5-S6	28	7.2
Tertiary	38	9.8
University	32	8.3
Occupation*	** **	
Unemployed	69	17.9
Self-employed	225	58.4
Salaried job	65	16.8
Other	26	6.7

*-missing data- 5 participants missing age, 2 missing occupation

+ _ Other on occupation included participants who sell second hand clothes, work in food catering, craft making, herbalists, land lords etc.

### Lifestyle and health related factors among study participants

Of the participants, 70.5% reported not drinking alcohol and 94.3% reported not smoking cigarettes in the past 12 months. The majority of the participants (94.8%) had WHO HIV staging of I, and 93.5% did not have an opportunistic infection as shown in Table 2.

**Table 2:** Lifestyle and health related factors among 387 participants at the Joint Clinical Research Centre, Lubowa March-April 2017.

Variable	Number (N = 387)	Percentage (%)
**Alcohol drinking**		
No	273	70.5
Yes	114	29.5
**Cigarette smoking**		
No	365	94.3
Yes	22	5.7
**WHO HIV stage**		
I	365	94.8
II	17	4.4
III	3	0.8
**Opportunistic infection**		
Yes	25	6.5
No	361	93.5

### Prevalence of NCDs among study participants

The overall prevalence of having at least one NCD was 20.7% (95% CI: 16.7–24.5). Prevalence was significantly higher among older participants, widowed participants and individuals with an opportunistic infection.

It was also slightly higher among males compared to females. However, there was no statistically significant difference by education, occupation, alcohol drinking, smoking and WHO HIV stage (Table 3).

**Table 3:** Prevalence of NCDs by socio demographic, lifestyle and health related factors among 387 participants at JCRC, March-April 2017.

Variable	Frequency N = (387)	NCD (n = 80)	Prevalence (%)	95% CI
**Age (categorized)**				
18–29	43	3	7.0	2.1–20.3
30–49	243	43	17.7	13.4–23.1
50–69	92	30	32.6	23.7–43.0
70 +	4	2	50	2.5–97.5
**Sex**				
Female	256	50	19.5	14.6–24.4
Male	131	30	22.9	15.6–30.2
**Marital status**				
Single	91	12	13.2	4.9–20.1
Married/Living together	185	39	21.1	14.1–26.0
Widowed	51	14	27.4	14.8–40.1
Divorced/Separated	60	15	25.0	13.7–36.3
**Alcohol drinking**				
No	273	54	19.8	15.0–24.5
Yes	114	26	22.8	15.0–30.6
**Cigarette Smoking**				
Yes	22	5	22.7	3.71–41.7
No	365	75	20.5	16.4–24.7
**Opportunistic infection**				
No	361	69	19.1	15.0–23.2
Yes	25	11	44.0	23.1–64.9

The most commonly observed NCD was hypertension at 12.4% (95% CI: 9.1–15.7), followed by osteoporosis at 6.5% (95% CI: 4.0–8.9) diabetes mellitus at 4.7% (95% CI: 2.6–6.8), renal impairment at 1.6% (95% CI: 0.3–2.8), asthma at 1.6% (95% CI: 0.3–2.8), and cardiomyopathy at 1.3% (95% CI: 0.2–2.4). Prevalence of multi-morbidity was 4.7% (95% CI: 2.6–6.8) (Table 4).

**Table 4:** Prevalence of NCDs, by type among 387 participants at JCRC, Lubowa, March-April 2017.

Type of NCD	Prevalence (%)	[95% CI]
Overall (1 NCD or more)	80 (20.7%)	16.7–24.8
Hypertension	48 (12.4%)	9.1–15.7
Osteoporosis	25 (6.5%)	4.0–8.9
Diabetes mellitus	18 (4.7%)	2.6–6.8
Renal impairment	6 (1.6%)	0.3–2.8
Asthma	6 (1.6%)	0.3–2.8
Cardiomyopathy	5 (1.3%)	0.2–2.4
Multi-morbidity(2 or more)	18 (4.7%)	2.6–6.8

## Discussion

Overall, 20.7% (80/387) of the participants had a non-communicable disease. These findings are similar with those of other studies carried out in Africa that show non-communicable diseases among people living with HIV range from about 15% to 58% [9–12]. However, these studies have looked at different NCDs with some NCDs such as: hypertension, diabetes mellitus, renal impairment, and asthma in common. A lot still needs to be done to give a clearer picture of the NCD co morbidity among PLHIV.

This is a high prevalence as it implies one in every five people has a non-communicable disease, yet is still an underestimate, as patient files were used and the actual prevalence is expected to be even higher.

Hypertension was the commonest NCD identified at 12.4% (95% CI: 9.1–15.7). This finding is similar to those from other studies among people living with HIV in Uganda and Africa [9, 13, 14]. A study carried out in rural Uganda among 65,000 people found a hypertension prevalence of 11% in people living with HIV and 14% in people without HIV. This is similar to our study findings and does not suggest differences between people with and without HIV [14, 15].

Osteoporosis was second highest at 6.5% (95% CI: 4.0–8.9) which is comparable to findings from a study carried out in Uganda at the Infectious Disease Institute among a cohort of people living with HIV receiving care at an outpatient HIV clinic which reported prevalence of osteoporosis of about 8 to 9% [16, 17]. This is a high percentage and osteoporosis diagnosis and treatment should be given more priority. Osteoporosis is known to occur in older HIV patients and menopausal women a group that is increasing in PLHIV[16].

Diabetes mellitus was found at 4.7% (2.5–6.8) which is similar to findings from studies among PLHIV in Africa which are mostly below 5% according to a systematic review by Haregu et al 2012. [6]. And it is also similar to that in the general community estimated to range from 1 to 4% [18]. However, other studies have reported type II diabetes as being more common among PLHIV [19, 20]. A study in people living with HIV in Nigeria 2016, reported prevalence of diabetes increasing from 2.3% to 5.3% in the first year of being started on ART[20].

Renal impairment was found at 1.6% (95% CI: 0.3–2.8), which is a low prevalence. However, this finding is similar to findings from a study based on self-reports, carried out in Nigeria [12]. However, this is a much lower prevalence than that from studies that actively screen for renal impairment. A study carried out in Burundi among PLHIV reported 30% as being stage I, 13.5% as stage II and 2% as being stage III [21]. Renal impairment is expected to be common, though it is rarely severe, among HIV-infected adults with clinically non-advanced HIV disease [22]. For most individuals it goes undiagnosed until it is severe.

Multi morbidity was found at 4.7% where by individuals had more than one non-communicable disease in addition to living with HIV. This finding is quite similar to that reported in Zimbabwe [9]where co-morbidity was found to be 4.5% (95% CI 3.4–6.0) considering hypertension, asthma, type 2 diabetes mellitus, cancer, and congestive cardiac impairment among PLHIV. However, since patients were not actively screened in this study the actual multi-morbidity prevalence is expected to be even higher than what is reported, and interventions targeting this group of people should be considered as challenges such as a high pill burden or higher costs of treatment are more common in this group.

Asthma and cardiomyopathy cases identified were very low at 1.6% and 1.2%. This maybe a result of not actively screening for these conditions; and is indicative of the fact that most patients do not know they have certain conditions until they are severe. In addition, this study was carried out at the free outpatient clinic and patients aware of such conditions are more likely to be in a more intensive care setting such as the inpatient clinic or private patients’ clinic.

Older people were found to be more likely to have a non-communicable disease (OR = 3.17, 95%CI: 1.87–5.41), which is expected as older people are known to have a much higher risk of non-communicable diseases compared to younger people [6, 9, 12]. Also, people with an opportunistic infection were more likely to have a non-communicable disease as well (OR = 3.33). This association has been identified by other studies as well according to a systematic review by Haregu T et al in 2012 who attributed it to NCDs complicating adherence to HIV treatment and also weakening patients’ immune systems hence increasing likelihood of opportunistic infections. Overall NCDs are prevalent among PLHIV, and strategies to encourage early diagnosis and treatment should be intensified.

### Strengths of the study

The study was conducted at an urban HIV specialized care centre that attends to needs of over 15,000 people living with HIV in Uganda. This is a large proportion of PLHIV in Uganda hence study findings are generalizable to all other PLHIV receiving care at an urban centre in the country.

Extraction of patient’s disease status/ patient information was done by qualified registered nurses currently working at JCRC that are familiar with HIV related research studies and have knowledge regarding non-communicable diseases. This greatly improved the quality of the data collected.

Routine care patient files were used as the primary data source for this study; this shows routinely collected data can be used to make significant findings that are valuable especially in resource limited settings such as Uganda.

No other studies in Uganda have looked at co-morbidity in relation to HIV while assessing several diseases yet a patient experiencing multi-morbidity has to live with several illnesses at the same time, not independently as how other studies only investigating a co-morbidity may imply. It gives a clearer image from the perspective of the patient.

This study highlights the need to not only focus on HIV but other chronic illnesses as well.

### Limitations

The study was conducted at only one site located in an urban area therefore the study findings may not be generalizable to people receiving HIV care from other sites that are rural or not specialized HIV care sites, or are government facilities.

In addition, patients at JCRC receive their medication on average every 3 months thus sampling from patients who attended the facility from March–April did not include some patients. However, there is no known distinction between the patients that come in the different months, so should not significantly affect the findings.

This study was carried out at the out -patient clinic where patients received free antiretroviral therapy, thus inpatients and private patients were not included in the study. However, it is expected that most inpatients may have a higher prevalence of co morbidities. More so patients with known co-morbidities may have been referred to other specialized care facilities.

The study population also had no individuals with HIV stage IV, so study findings are not entirely generalizable to all individuals receiving antiretroviral therapy.

This study also had the limitation of using patient files to determine if an individual has an NCD or not. As much as this gives an estimate of the prevalence of NCDs, the actual NCD burden is higher than what this study estimates. Thus the results need to be interpreted with that in consideration.

## Conclusions

Non-communicable diseases are a growing concern not only due to their health implications, but also their impact on socio economic status. This study found that 1 in every 5 people living with HIV, has an NCD. There is need to encourage early diagnosis and treatment of non-communicable diseases among PLHIV in Uganda. More research is also still needed to describe the co morbidity burden in PLHIV even further.

## Supporting information

S1 Questionnaire S1 English screening log, questionnaire, consent forms. (PDF)

S2 Questionnaire S2 Alternative Language consent form, questionnaire. (PDF)
