# Supplementary material for: Prevalence of non-communicable diseases among HIV positive patients on antiretroviral therapy at joint clinical research centre, Lubowa, Uganda
Source: PLoS One. 2019 Aug 9;14(8):e0221022. doi: 10.1371/journal.pone.0221022 (PMC6688817; doi:10.1371/journal.pone.0221022)
Supplement: S1 Questionnaire — (PDF) [file pone.0221022.s001.pdf]

Appendix 1: Participants screening log

Study title: <u>Non-communicable diseases and their association with quality of life among patients on art at JCRC, Lubowa</u>

Investigator: ___________________________

Participant ID #: ______________ Date: ___/___/_____

ELIGIBILITY CRITERIA CHECKLIST

INCLUSION	EXCLUSION
All boxes must be checked **Yes** for Participant to be considered eligible<br>Age; (16 years and above). □Yes □No<br><br>Receiving ART □ Yes □No<br><br>Giving informed consent □Yes □No	All boxes must be checked **No** for Participant to be considered eligible<br>Pregnant □Yes □No □N/A<br>physically and/ or mentally unable to participate □ Yes □No<br>Unable to comprehend either English, Luganda □Yes □No

I have reviewed this Participant's medical information and have determined that they have met the criteria for eligibility into the above-mentioned study.

Completed by ___________________________ ___/___/______ _________

Person Verifying Eligibility Date Time

Information Reviewed by ___________________________ ___/___/______

Principal Investigator Date

Appendix 2: Questionnaire

STUDY TITLE: NON-COMMUNICABLE DISEASES AND THEIR ASSOCIATION WITH QUALITY OF LIFE AMONG PATIENTS ON ART AT JCRC, LUBOWA

Study ID NO___________ Date of interview: _____/_____/_____

A. Socio-Demographic characteristics

1. Age (in complete years):____________

2. Sex: □1=Male □2=Female

3. Religion: □1=Protestant □2=Catholic □3=Moslem

□4= born-again □5=others (specify) ________________

4. Marital status: □1=Single □2=Married/Living together □3= Never married

□4= Widowed □5=Divorced/Separated □6=others (specify) __________

5 . Highest level of education:

□1=No formal education □2=Primary □3=S1-S4 □4=S5-S6 □5=Tertiary □6=University

6. Occupation: □1=Un-employed □2=Self-employed (specify)______________ □3=salaried job (specify)___________________

B. Lifestyle questions

7. How often do you take an alcoholic drink?

□1 = I don't drink anything alcoholic □2= Once a week

□3= 2-4 times a week □4=every day of the week □5= Once a month

8. Which type of alcoholic drink do you usually take?

□1= Wine □2= Beer

□3= Spirits/waragi/gin/whisky/vodka □4= Local brew (specify) _____________

9. Have you smoked cigarettes any time in the past 12 months?

□1= Yes □2= No

10. IF yes, in the last 1 week how many cigarettes did you smoke? _____________

C. Other health related factors

11. WHO HIV stage _____________________

12. Opportunistic infections

□1= Yes □2= No

D. Non Communicable Diseases (to be extracted from the patient files)

Please tick if patient has a given disease:

13. Hypertension □1= Yes □2= No

14. Diabetes mellitus □1= Yes □2= No

15. Renal impairment □1= Yes □2= No

16. Asthma □1= Yes □2= No

17. Cardiovascular diseases □1= Yes □2= No

If yes specify ______________________________

18. Osteoporosis □1= Yes □2= No

19. Cancers □1= Yes □2= No

If yes specify ________________________________

Appendix 3: Consent/ Assent form

STUDY TITLE: NON-COMMUNICABLE DISEASES AND THEIR ASSOCIATION WITH QUALITY OF LIFE AMONG PATIENTS ON ART AT JCRC, LUBOWA

INTRODUCTION

This research study is being done by Kansiime Sheila, a Master's of science in clinical epidemiology and Biostatistics student at Makerere University. Before you decide if you want to participate, we would like you to know more about the study.

This is a consent form. It gives information about this study. If you are willing to participate, we will ask you to sign this consent form. You will get a copy of this form to keep as well.

Why is this study being done?

Non communicable diseases are those diseases not passed on from person to person, are of long duration, and of generally slow progression. They include hypertension, diabetes mellitus, cardiovascular diseases, Asthma, cancers, and renal failures . They are mainly caused by four main risk factors; Tobacco use, physical inactivity, unhealthy diets, and alcohol use, in addition to other factors. They are on the rise internationally and are responsible for a lot of death and disability. Among people living with HIV, some of these illnesses have been identified as occurring more commonly than in the general population. However in Uganda information on how common they are is still lacking. In 2012 CDC recommended HIV-NCD care models be integrated to address this issue, however information from developing countries guiding the model creation is still lacking.

This study aims to determine the prevalence of non-communicable diseases (focusing on hypertension, diabetes mellitus, asthma, renal impairment, cardiovascular diseases, osteoporosis and cancers) and their association with health related quality of life in HIV positive people in Kampala, Uganda. This study also aims to identify what challenges are encountered by PLHIV in seeking for healthcare services for the non communicable diseases.

What will happen if you are part of this study?

If you decide to take part in this study, you will be part of 400 participants recruited from Joint clinical research centre. With your permission details about which non-communicable diseases you may be having will be retrieved from your patient file. You will then take part in an interview which will take 15-20 minutes.

What are the possible benefits from being in this study?

The main benefit of participating will be being involved in a study that will contribute to knowledge on how to address the issue of non-communicable diseases among people living with HIV. Its studies like this that can guide policy makers on how to address health issues.

What are the possible risks?

You may feel uncomfortable providing information about you to strangers and the interview may take up 15-20 minutes of your time.

How will your privacy be ensured?

All information which will be collected from you during the course of this research will be kept strictly confidential. All records will be kept in a safety cabinet, under lock and key.

Your name will not be extracted from your patient file or taken during interview, as your information in this study will be assigned a PIN.

Your name may only be taken on this consent form, which will stay kept within joint clinical research center.

Rights to decline or withdraw from the study

Participation in research is completely voluntary. You may refuse to participate in this study or withdraw your consent at any time for any reason and this will not in any way affect how you receive health care services from this facility or any other.

WHOM TO CONTACT:

If you have any questions, complaints or concerns regarding this study, you can contact the investigator:

Kansiime Sheila on mobile phone number 0779113154

If you have any questions regarding the rights or any other ethical issues concerning your participation in this study, you may contact Prof. Ponsiano Ocama, chairman School of Medicine Research and Ethics Committee on mobile phone number;0414530020

Statement of consent:

The investigator has described to me what is required of me as a participant, the procedures, the risks involved, the benefits, and my rights regarding the study. I have been informed that the information given will be kept confidential and that my participation in this study is voluntary and that no consequences will result if I decline to participate or withdraw from the study.

I understand that by signing this form, I do not waive any of my legal rights but merely indicate that I have been informed about the research study in which I am voluntarily agreeing to participate.

Name:..signature of participant...............................date.......................

Name:..signature of interviewer:..............................date.....................
