# Supplementary material for: Prevalence of non-communicable diseases among HIV positive patients on antiretroviral therapy at joint clinical research centre, Lubowa, Uganda
Source: PLoS One. 2019 Aug 9;14(8):e0221022. doi: 10.1371/journal.pone.0221022 (PMC6688817; doi:10.1371/journal.pone.0221022)
Supplement: S2 Questionnaire — (PDF) [file pone.0221022.s002.pdf]

# S2 Alternative Language (Luganda) consent form and questionnaire

## Omutwe gwokunoonyereza: endwadde zolukonvuba wamu nenkwatagana yaazo n'omutindo gwobulamu mu balwadde abali ku ddagala lya ssiriimu ku kitongole kya 'JCRC' lubowa.

**Enyanjula :**

Okunoonyereza kuno kukolebwa Kansiime Sheila, omuyizi wa ddiguli eyokubiri mu kitongole kya 'Clinical Epidemiology and Biostatistics' ku setendekero ya Makerere. Nga tonnaba kusalawo kwetaba mu kunoonyeereza,twandyagadde omanye bingi ebilala ebikwata ku kunoonyereza.

Kino kiwandiiko kya kukkiriza kwetaba mu kunoonyereza. Kiwa obubaka obukwata ku kunoonyereza. Bwoba oyagala okwetabamu, tujja kukusaba oteeke omukono ku kiwandiiko. Ojja kufuna kkopi yekiwandiiko ojiteleke.

**Okunoonyereza kuno lwaki kukolwa?**

Endwadde ezolukonvuba zeezo ezitasaasanyizibwa kuva ku muntu omu okudda ku mulala,za bbanga ppanvu,era nga zikula mpola. Mulimu ppuleesa, ssukaali, obulwadde bwomutima, asima, kookolo,wamu nendwadde zakawago. Zisinga kuleetebwa mbeera ezakatyabaga nga; Okukozesa taaba, obutazanya, okulya obubi, wamu nokukozesa omwenge, nga kwogasse embeera endala. Zilinye ku daala lya nsi yonna era ziviiliddeko okufa kunji wamu nokulemala. Mu bantu ababeera nakawuka ka ssiriimu, endwadde zino ezimu zilabiddwa nga zibaawo emirundi minji okusinga mu bantu abalala. Wabula, mu Uganda obubaka obukwata ku bungi bwazo tebunnalabika. Mu mwaka nga 2012, ekitongole kya CDC, kyalagila nti enteekateeka

ezikwata kugonjoola ekizibu kyendwadde eziva ku bulwadde bwa ssiriimu ziteekebwe mu nkola,wabula obubaka obuva mu mawanga agakyakula ogayamba ku kutondawo enkola gakyabuze.

Okunoonyereza kuno kugenderera okuzuula omuwendo gwendwadde ezolukonvuba (nga tuteeka essila ku ppuleesa, ssukaali,asima, okufa akawago, obulwadde bwomutima, wamu ne kookolo) wamu nenkolagana yaabwo n'omutindo gwobulamu mu bantu abakulu abalwadde ba ssiriimu mu Kampala, Uganda. Okunoonyereza kuno era kugenderera okuzuula ebizibu ebifunibwa abantu abalina akawuka ka ssiriimu mu kufuna obujanjabi ku lwobulwadde obwolukonvuba.

**<u>Kiki ekinaabawo singa weetaba mu kunoonyereza kuno?</u>**

Bwosalawo okwetaba mu kunoonyereza kuno, ojja kubeera omu ku beetabyemu 400 abali mu kitongole kya 'Joint Clinical Research Centre'. Wamu n'olukusalwo,obubaka obukwata ku bulwadde bwolukonvuba bwolina bujja kujjibwa mu biwandiiko byo ebyobulwadde. Olwo olyoke wenyigire mu bibuuzo ebinaatwala eddakiika wakati wamakumi abari n'asatu (15-20)

**<u>Byakuganyurwaki ebiri mu kunoonyereza kuno?</u>**

Ekyokuganyurwa ekikulu ekiri mu kwetaba mu kunoonyereza kwekwongera ku magezi ku ngeriki eyokukwatamu ensonga y'endwadde ezolukonvuba mu bantu abalina obulwadde bwa ssiriimu. Okunoonyereza bwekuti kwekuyinza okulagilira abakozi baamateeka ku ngeriki eyokukwatamu ensonga zebyobulamu.

**<u>Byakatyabagaki ebilimu.</u>**

Oyinza obutawulira bulungi ng'otuwa obubaka obukukwatako eri abantu bootamanyi era okubuuza ebibuuzo kuyinza okutwala eddakiika wakati wekkumi netaano nabiri ku budde bwo.

**Ebyama byo binaakumibwa bitya?**

Obubaka bwonna obunaakunganyizibwanga okuva kugwe mu kunoonyereza bijja kukuumibwa nga byakyaama. Ebikukwatako byonna bijja kutelekebwa ku kabada zisibibwe nekkufulu wamu nekisumuluzo.

Erinyalyo telijja kujjibwa mu biwandiiko byo ebyobulwadde oba litwalibwe mu kubuuza ebibuuzo,engeri obubakabwo mu kunoonyereza kuno gyebunaabera nennamba.

Erinyalyo liyinza okutwalibwa ku kiwandiiko kino kyokka,ekinaasigala ku 'Joint Clinical Research Centre'

Eddembe okuva mu kunoonyereza

**Okwetaba mu kunoonyereza**

Okwetaba mu kunoonyereza kuno kwakyeyagalire. Oyinza okugaana okwetaba mu kunoonyereza kuno oba okuvaamu akadde konna kulwensonga yonna era kino tekijja kukosa ndabilila yo gyofuna okuva ku dwaliro lino oba eddala.

**Ani owookutuukilira**

Bwoba olina ebibuuzo, ebyokwemulugunya, ebikwata ku kunoonyereza kuno, oyinza okutuukilira akulira okunoonyereza:

Kansiime Sheila ku ssimu 0779113154

Bwoba olina ebibuuzo ebikwata ku kunoonyereza oba ensonga endala ezikwata kukwetabakwo mu kunoonyereza, oyinza okutuukirira sabakenkufu Ponsiano Ocama, sentebe wakakiiko akakwasisa empisa mu kunoonyereza mu tendekero lyebyobulamu ku ssimu 0772421190.

**Olunyiliri lwokukkiriza**

Anoonyereza anyinyonyodde ekineetaagisa ng'eyetabye mu kunoonyereza, emitendera, ebyobutyabaga, neddembe lyange ebikwata ku kunoonyereza. Ntegeezedwa nti obubaka bwempa bujja kukuumibwa nga bwakyama era nti okwetabaamu kwange kwa kyeyagalire era nti tewali biva mu kugaana kwetabamu oba kuva mu kunoonyereza.

Ntegeera nti okuteeka omukono ku kiwandiiko kino,sikugira ddembe lyange lyonna lyabwembanje wabula k,ilaga nti ntegeezedwa ku kunoonyereza kweneeyagalidde okukkiriza okwetabamu.

…………………………………………………………

…………………………………………………… ………………………………...

Erinya lyeyetabyemu Omukono Enaku zomwezi

………………………………………………………….

…………………………………………………….

……………………………………

Erinya lyabuuza Omukono Enaku zomwezi

# Appendix 6: Luganda questionaire

**Omutwe gwokunoonyereza:obungi bwendwadde zolukonvuba wamu nenkwatagana yaazo n'omutindo gwobulamu mu balwadde abali ku ddagala lya ssiriimu ku kitongole kya 'jcrc' lubowa.**

**Study ID NO___________** Ennaku zo'mwezi: _____/_____/_____

**A. Ebikwata kubulamu bwo**

1. Oyina emyaka emeka (emijuvu)? ____________

2. Obutonde? □1=Mwami □2=Mukyala

3. Oli wa nzikiriza ki?

□1=Mukurisitayo □2=Mukaturiki □3=Musiramu □4= Mulokole

□5= Endala (nzikiriza ki?) ________________

4. Oli mufumbo?

□1=Ndi nzeka □2=mufumbo/mubrera mwembi □3= Sifumbirangako

5. Wasoma kutuuka wa?

□1=Sisomangako□2=Mukisooka pakaku kyomusanvu □3=Siniya esooka pakaku yokuna

□4= Siniya eyokutano pakaku yomukaaga □5= Mu tekiniko □6=Yunivasite

6. Okola mirimu ki? □1=Sikola □2=Nekozesa (murimu ki?)______________

□3= gwa buyigirize (murimu ki?)______________

**<u>E. Ebikwata ku kunywa ebitamiiza nokukozesa ebilagalagala</u>**

7 . Oteraddi okunywa kubitamiiza? *(If 1, skip Qn.29)*

□1 = Sinywa kintu kyona kitamiiza □2= Omurundi gumu muwiiki □3= Emirundi ebiri oba ena muwiiki □4=Bulu lunaku lwa wiiki □5= Omurundi gumu mumwezi

8 . Bitamiizaki kika kyi byoteera okunywa?

□1= Wayini □2= Biya

□3= Waragi/gin/whisky/vodka □4= Omwenge omuganda (kika ki?) _____________

9. Wali onywedde kusigala mu mwaka oguyise emabega?

□1= Yee □2= Nedda

10. Obba nga okiriiza, wanywa sigala nga meka mu wiki ewedde? _________________

**C. Other health related factors** (to be extracted from the patient files)

11. WHO HIV stage _____________________

12. Opportunistic infections

□1= Yes □2= No

**D. Non Communicable Diseases** (to be extracted from the patient files)

Please tick if patient has a given disease:

13. Hypertension □1= Yes □2= No

14. Diabetes mellitus □1= Yes □2= No

15. Renal impairment □1= Yes □2= No

16. Asthma □1= Yes □2= No

17. Cardiovascular diseases □1= Yes □2= No

If yes specify ________________________________

18. Osteoporosis □1= Yes □2= No

19. Cancers □1= Yes □2= No

If yes specify ______________________________
